# Efficient genetic transformation and gene editing of Chinese cabbage using *Agrobacterium rhizogenes*

**DOI:** 10.1093/plphys/kiae543

**Published:** 2024-10-15

**Authors:** Yaolong Wang, Xuedong Yang, Wenlong Wang, Yan Wang, Xiaoshan Chen, Han Wu, Zhanyuan Gao, Huanhuan Xu, Tongkun Liu, Ying Li, Dong Xiao, Wusheng Liu, Xilin Hou, Changwei Zhang

**Affiliations:** National Key Laboratory of Crop Genetics and Germplasm Enhancement and Utilization, Nanjing Agricultural University, Nanjing 210095, China; Shanghai Key Laboratory of Facility Horticulture Technology, Shanghai Academy of Agricultural Sciences, Shanghai 200062, China; National Key Laboratory of Crop Genetics and Germplasm Enhancement and Utilization, Nanjing Agricultural University, Nanjing 210095, China; National Key Laboratory of Crop Genetics and Germplasm Enhancement and Utilization, Nanjing Agricultural University, Nanjing 210095, China; National Key Laboratory of Crop Genetics and Germplasm Enhancement and Utilization, Nanjing Agricultural University, Nanjing 210095, China; National Key Laboratory of Crop Genetics and Germplasm Enhancement and Utilization, Nanjing Agricultural University, Nanjing 210095, China; National Key Laboratory of Crop Genetics and Germplasm Enhancement and Utilization, Nanjing Agricultural University, Nanjing 210095, China; Shanghai Key Laboratory of Facility Horticulture Technology, Shanghai Academy of Agricultural Sciences, Shanghai 200062, China; National Key Laboratory of Crop Genetics and Germplasm Enhancement and Utilization, Nanjing Agricultural University, Nanjing 210095, China; National Key Laboratory of Crop Genetics and Germplasm Enhancement and Utilization, Nanjing Agricultural University, Nanjing 210095, China; National Key Laboratory of Crop Genetics and Germplasm Enhancement and Utilization, Nanjing Agricultural University, Nanjing 210095, China; National Key Laboratory of Crop Genetics and Germplasm Enhancement and Utilization, Nanjing Agricultural University, Nanjing 210095, China; Department of Horticultural Science, North Carolina State University, Raleigh, NC 27695-7609, USA; National Key Laboratory of Crop Genetics and Germplasm Enhancement and Utilization, Nanjing Agricultural University, Nanjing 210095, China; Nanjing Suman Plasma Engineering Research Institute Co., Ltd., Nanjing 211162, China; National Key Laboratory of Crop Genetics and Germplasm Enhancement and Utilization, Nanjing Agricultural University, Nanjing 210095, China

## Abstract

A method using *Agrobacterium rhizogenes*-mediated callus production and plant regeneration enables efficient genetic transformation and gene editing in Chinese cabbage.

Dear Editor,

Crop genetic transformation and regeneration is a crucial step in the CRISPR/Cas-based genome editing for functional analysis and crop trait improvement (Maren et al. 2022; Ramasamy et al. 2023). The low *Agrobacterium tumefaciens*-mediated transformation efficiency limits the use of gene editing in Chinese cabbage (*Brassica rapa*), a widely cultivated vegetable crop (Jeong et al. 2019). Recently, the use of growth and developmental regulator genes together with *A. tumefaciens*-mediated transformation has been employed to enhance the efficiency of crop transformation and the regeneration of fertile plants. For example, the *WUSCHEL* (*WUS*), *ISOPENTENYL TRANSFERASE* (*IPT*), or *PLETHORA5* (*PLT5*) genes have successfully been used to facilitate genetic transformation in various crops, such as maize (*Zea mays*), rice (*Oryza sativa*), tomato (*Solanum lycopersicum*), sweet pepper (*Capsicum annum*), and Bok choy and Pai-Tsai (*B. rapa*), etc. (Lowe et al. 2016; Maher et al. 2020; Lian et al. 2022).

In addition, *A. rhizogenes*-mediated genetic transformation has largely been used for the regeneration of transgenic hairy roots rather than transgenic plants since it is extremely challenging to regenerate shoots from those transgenic roots of many plant species including Chinese cabbage. Recent breakthroughs found that exogenously applied hormones have been successfully utilized to regenerate shoots directly from the transgenic hairy roots obtained through *A. rhizogenes*-mediated transformation in oilseed rape (*B. napus*), apple (*Malus domestica*), kiwifruit (*Actinidia chinensis*), and citrus (Jedlickova et al. 2022; Ramasamy et al. 2023; Liu et al. 2024). However, the success in shoot regeneration from the transgenic hairy roots largely rely on the crop capacity for the hairy root-to-shoot conversion and the tedious tests with hormone types and concentrations in each crop, limiting the application of the method to other crops. In the present study, we established an efficient genetic transformation and gene editing method in Chinese cabbage by regenerating transgenic and gene-edited plants from the transgenic calli produced by *A. rhizogenes*-mediated transformation without the use of exogenously applied hormones for shoot regeneration (Fig. 1). This was achieved with the help of growth and developmental regulator genes (Fig. 1).

**Figure 1.**
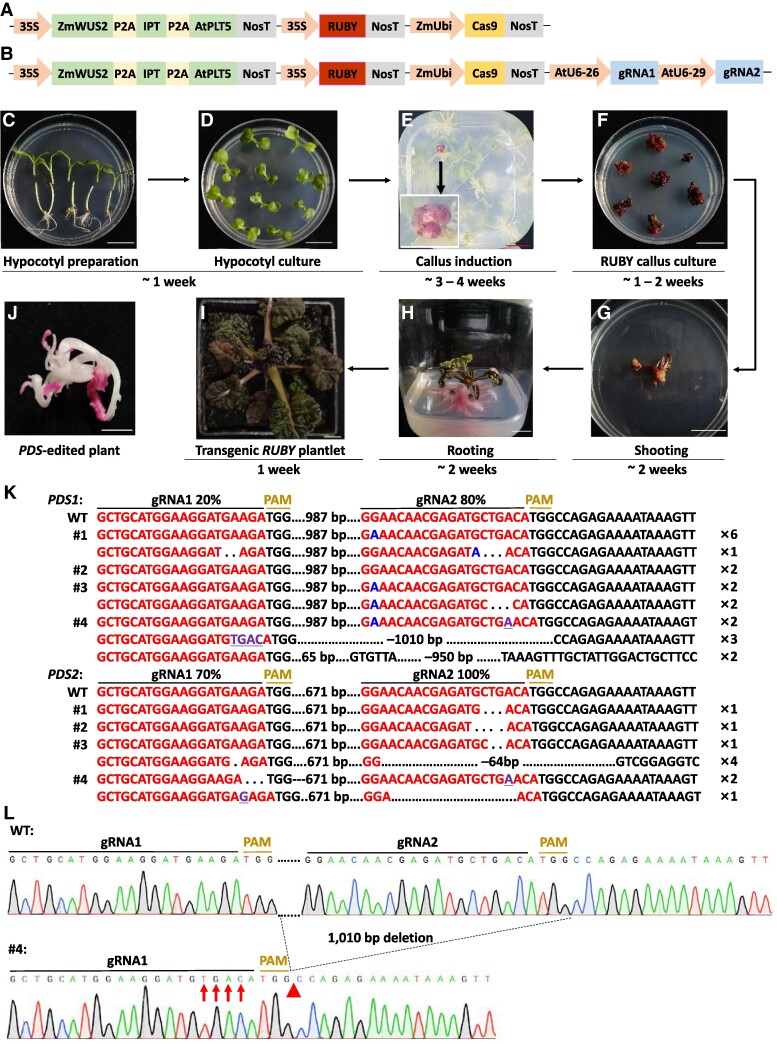
*A. rhizogenes*-mediated callus induction and transgenic plant regeneration for genetic engineering and gene editing in Chinese cabbage. **A)** The plasmid used for Chinese cabbage transformation and containing *35S*:*ZmWUS2-IPT-AtPLT5*, which were linked with the “self-cleaving” P2A sequence, *35S:RUBY*, and *AtUbi:Cas9*. The *IPT* gene was from *Agrobacterium*. **B)** The plasmid used for Chinese cabbage gene editing and containing the genes in (A) and the *AtU6-26:gRNA1*-*AtU6-29:gRNA2* targeting the *PDS1* and *PDS2* genes in Chinese cabbage simultaneously. **C** to **I**) The flowchart of our *A. rhizogenes*-mediated callus induction and transgenic plant regeneration approach in Chinese cabbage, which takes about 3 months. Scale bar = 20 mm. **J)** Representative image of the edited *pds* mutant plants of Chinese cabbage. Scale bar = 10 mm. **K)** DNA sequencing results showing targeted mutations in the edited *pds1* and *pds2* mutants of Chinese cabbage. Dot, nucleotide deletion; purple and underline, nucleotide insertion; blue, nucleotide substitution; red, target site; WT, cv. “49Caixin” wild type; PAM, protospacer adjacent motif. The number of single colonies showing each type of mutations after Sanger sequencing was indicated at the right as “×n”. **L)** Sanger sequencing result showing the targeted mutation in the *PDS1* gene in Line #4. Red arrows, nucleotide insertion; red triangles, nucleotide deletion.

We cloned *35S:RUBY* (He et al. 2020), *35S:ZmWUS2*-*IPT*-*AtPLT5* where the “self-cleaving” P2A sequence was used to link the three growth and developmental regulator genes together, and *ZmUbi:Cas9*—*AtU6:gRNAs* into a binary plasmid (Fig. 1A). The resultant destination plasmid was confirmed by Sanger sequencing and introduced into *A. rhizogenes* (K599), followed by transformation into 5- or 6-day-old seedlings of the elite, nonheading Chinese cabbage cv. “49Caixin.” The hypocotyl explants, which were still attached to cotyledons (Fig. 1C), were co-cultivated with a suspension of *A. rhizogenes* (OD_600_ = 0.8) carrying the destination plasmid for 10 min. The explants were transferred to the Murashige and Skoog solid medium (pH 5.8) containing 0.9% agar, 3% sucrose, and 200 mg/L carbenicillin and cultured under a photoperiod of 16 h light and 8 h dark (Fig. 1D, Supplementary Materials and Methods). Roots and calli were developed near the incision sites of the explants after 15–25 days (Fig. 1E). We used 100 explants per replicate for three replicates of the experiments, and found that some of the explants developed red-colored roots and calli due to the heterologous overexpression of the *RUBY* gene, which was used as a visual reporter (Fig. 1E). The presence of the transgenes in the red calli were confirmed by PCR amplification of the *Cas9* gene (Supplementary Fig. S1) using the gene-specific primers (Supplementary Table S1). The transgenic callus induction frequency, which was calculated by dividing the number of transgenic calli by the total number of explants, was 20.48% (Supplementary Table S2).

After the roots were removed from the embryogenic red calli, the calli were cultured on the above-mentioned media (Fig. 1F) and shoots with shoot apical meristems and a normal phenotype were regenerated from the calli (Fig. 1G). We did observe abnormal leaves and shoots after regeneration (Supplementary Fig. S2) due to the continual ectopic expression of the growth and developmental regulator genes as reported by Lian et al. (2022), justifying the use of inducible expression of these genes (e.g. using heat-inducible promoters) for future cultivar development. This shoot regeneration from the transgenic calli was achieved without the use of hormones in the media. We found that 2 ∼ 3 shoots could be obtained from a single piece of embryogenic red callus, and about 90% of the shoots were red colored while the remaining 10% shoots were green, indicating the heterogeneity of the calli (Supplementary Fig. S2). The heterogeneity of the calli could result from the effects of the growth and development regulators on the untransformed cells surrounding the transformed cells. When the regenerated shoots were cut off from the red calli and cultured on the root induction media, the red shoots developed red roots (Fig. 1H). After being transplanted to soil, the red plantlets developed into mature plants with red wrinkled leaves and more branches when compared to the wild-type plants (Fig. 1I; Supplementary Fig. S3, A and B). Transgenic plants were fertile and produced normal quantity of fertile seeds (Supplementary Fig. S3, C to E). Thus, we have successfully established a transformation method in cv. “49Caixin” via *A. rhizogenes*-mediated callus induction and plant regeneration (Fig. 1).

For comparison, the resultant destination plasmid was also introduced into *A. tumefaciens* (GV3101) and used for cv. “49Caixin” transformation. However, we did not find any red calli or shoots developed from the explants after *A. tumefaciens* transformation (Supplementary Fig. S4). Thus, the use of *A. rhizogenes* was critical for the success of this transformation method.

To assess the feasibility of this method in different Chinese cabbage cultivars, we used this method for transgenic callus induction and shoot regeneration in seven more elite Chinese cabbage cultivars, i.e. the nonheading cultivars “Suzhouqing,” “Aijiaohuang,” “Shanghaiqing,” and “Huangmeigui,” and the heading cultivars “Bre,” “082,” and “Chiifu” (Supplementary Fig. S5). We found that the red, embryogenic callus induction frequency ranged from 3.31% to 15.55% in these cultivars (Supplementary Table S2), demonstrating the genotype independence of our transformation method. The success in the transformation of cvs. “Suzhouqing” and “Chiifu” holds particularly profound implications for gene function analysis and gene editing research because both cultivars were used as reference genomes.

We also extended our transformation method for gene editing of the *PHYTOENE DESATURASE* (*PDS*) gene in cv. “49Caixin.” *PDS* is a carotenoid biosynthesis pathway gene and has two homologs, i.e. *PDS1* (*Bra032770*) and *PDS2* (*Bra010751*) in Chinese cabbage due to the whole genome triplication (Wang et al. 2011). We designed two gRNA target sites to target both homologs simultaneously (Supplementary Table S1; Supplementary Fig. S6), which were cloned into the destination plasmid used for the above-mentioned genetic transformation of Chinese cabbage (Fig. 1B). Out of 100 explants, 11 transgenic calli were successfully obtained and 7 albino plants were regenerated (Fig. 1J). We used PCR and Sanger sequencing to confirm the gene editing of the *PDS* genes in 4 out of the 7 albino regenerated plants (Supplementary Materials and Methods), which were named Lines #1 to #4 (Supplementary Table S1). Upon identification of double peaks at the target sites, mutation types and frequencies were analyzed by performing TA cloning and Sanger sequencing of the PCR products. The majority of the mutations consisted of substitutions, short insertions, or deletions (Fig. 1K; Supplementary Figs. S7 and S8). Notably, deletions of 950 bp and 1,010 bp in length were observed in the *PDS1* gene in Line #4 (Fig. 1L). The highest mutation frequency was 100% at the target site 2 of *PDS2*, followed by 80% at the target site 2 of *PDS1*, and 70% at the target site 1 of *PDS2*, while the target site 1 of *PDS1* had the lowest mutation frequency (20%; Fig. 1K). As a result, all of these data confirmed the feasibility of our transformation method for gene editing in Chinese cabbage.

In summary, we have successfully established a simplified and efficient genetic transformation and gene editing system in Chinese cabbage without the need to use *A. tumefaciens* for transformation or hormones for shoot regeneration. The whole process from seed germination to callus induction, shooting, and rooting can be completed within three months (Fig. 1, C to I). This transformation method can overcome the genotype dependence of genetic transformation of Chinese cabbage cultivars, holding great promise for functional genomics studies, gene editing, and crop improvement in elite Chinese cabbage cultivars.

## Supplementary Material

kiae543_Supplementary_Data

## Data Availability

The data underlying this article are available in the article and in its online supplementary material.

## Supplementary data

The following materials are available in the online version of this article.

**Supplementary Table S1.** The primers used in the present study.

**Supplementary Table S2.** The positive callus induction frequency in the eight Chinese cabbage cultivars.

**Supplementary Figure S1.** Confirming the presence of the *Cas9* transgene in the *A. rhizogenes*-mediated transgenic cv. “49Caixin”.

**Supplementary Figure S2.** Shoot regeneration from the red callus of the *A. rhizogenes*-mediated transgenic cv. “49Caixin”.

**Supplementary Figure S3.** The representative *A. rhizogenes*-mediated transgenic cv. “49Caixin” overexpressing *RUBY*.

**Supplementary Figure S4.**  *A. tumefaciens*-mediated transformation of the cv. “49Caixin”.

**Supplementary Figure S5.** Induction of *A. rhizogenes*-mediated transgenic calli in the eight Chinese cabbage cultivars after the hypocotyls were cut from roots and cultured together with leaves.

**Supplementary Figure S6.** The genomic DNA sequences of the *PDS1* (*Bra032770*) and *PDS2* (*Bra010751*) genes in the *B. rapa* reference genome (cv. Chiifu V1.5).

**Supplementary Figure S7.** Confirming the editing-induced mutations at the two gRNA target sties in *PDS1* in the four albino edited plants (Lines #1 to #4) of cv. “49Caixin”.

**Supplementary Figure S8.** Confirming the editing-induced mutations at the two gRNA target sties in *PDS2* in the four albino edited plants (Lines #1 to #4) of cv. “49Caixin”.

## Dive Curated Terms

The following phenotypic, genotypic, and functional terms are of significance to the work described in this paper:

AtPLT5 Gramene: AT3G18830
AtPLT5 Araport: AT3G18830

PDS1 Gramene: AT1G06570
PDS1 Araport: AT1G06570

phytoene CHEBI: CHEBI:27787
