# Supplementary material for: Efficient genetic transformation and gene editing of Chinese cabbage using Agrobacterium rhizogenes
Source: Plant Physiol. 2024 Oct 15;197(2):kiae543. doi: 10.1093/plphys/kiae543 (PMC11849774; doi:10.1093/plphys/kiae543)
Supplement: kiae543_Supplementary_Data [file kiae543_supplementary_data.zip › Supplementary Data.pdf]

Supplementary Tables

Supplementary Table S1. The primers used in the present study

**Primer Name**	**Primer Sequence (5' > 3')**
gRNA target sites:	
sgRNA1	GCTGCATGGAAGGATGAAGA
sgRNA2	GAAACAACGAGATGCTGACA
Detection of edited mutations:	
Bra032770-F	AGTTGTCATTGCTGGTGCTGGT
Bra032770-R	GGAGTGAGCATGCTTGAGTGGT
Bra010751-F	TTTCCGTAGTGCTCCTCGTCCT
Bra010751-R	AGCACACGAGAAGGAAGCAACA
Confirmation of the presence of the transgene in the transgenic lines:	
Cas9-F	GACAAGAAGTACTCCATCGG
Cas9-R	CTCGATCTTCTTGAAGTAGT

The genes' information (*Bra032770*, *Bra010751*) comes from Brara_Chiifu_V1.5 genome version (http://brassicadb.cn).

Supplementary Table S2. The positive callus induction frequency in the 8 Chinese cabbage cultivars

Cultivars	Replicate	Explant #	*RUBY*-Positive Callus #	Positive Callus Induction Frequency (%)	Average Efficiency (%)
49Caixin	1	295	63	21.36	20.48 ± 2.54
	2	325	73	22.46	
	3	335	59	17.61	
Suzhouqing	1	190	20	10.53	10.10 ± 1.14
	2	200	21	10.50	
	3	105	9	8.57	
Aijiaohuang	1	51	5	9.80	11.24 ± 1.21
	2	60	7	11.67	
	3	58	7	12.07	
Shanghaiqing	1	58	3	5.17	5.95 ± 0.67
	2	65	4	6.15	
	3	62	4	6.45	
Huangmeigu	1	102	16	15.69	15.55 ± 1.29
	2	98	14	16.87	
	3	83	14	14.29	
Bre	1	64	8	12.50	13.27 ± 0.70
	2	60	8	13.33	
	3	72	10	13.89	
082	1	104	3	2.88	3.31 ± 0.57
	2	97	3	3.09	

Table S2. (Cont.)

Cultivars	Replicate	Explant #	*RUBY*-Positive Callus #	Callus Induction Frequency (%)	Average Efficiency (%)
	3	101	4	3.96	
Chiifu	1	57	5	8.77	8.74 ± 0.52
	2	65	6	9.23	
	3	61	5	8.20	

The callus induction frequency was calculated by dividing the number of red callus by the total number of explants.

Supplementary Figures

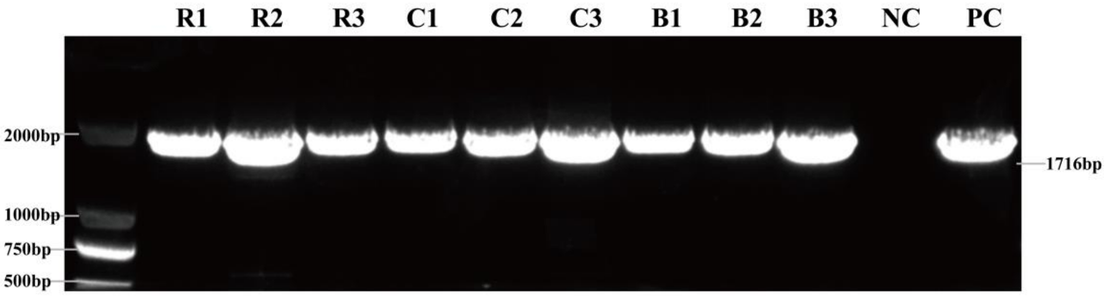

Supplementary Figure S1. Confirming of the presence of the transgene *Cas9* in the *A. rhizogenes*-mediated transgenic cv. '49Caixin'. R1 – R3, *RUBY* hairy roots; C1 – C3, *RUBY* calli; B1 – B3, *RUBY* shoots. The wild-type genomic DNA of cv. 49Caixin was used as the negative control (NC) while the plasmid DNA were used as the positive control (PC).

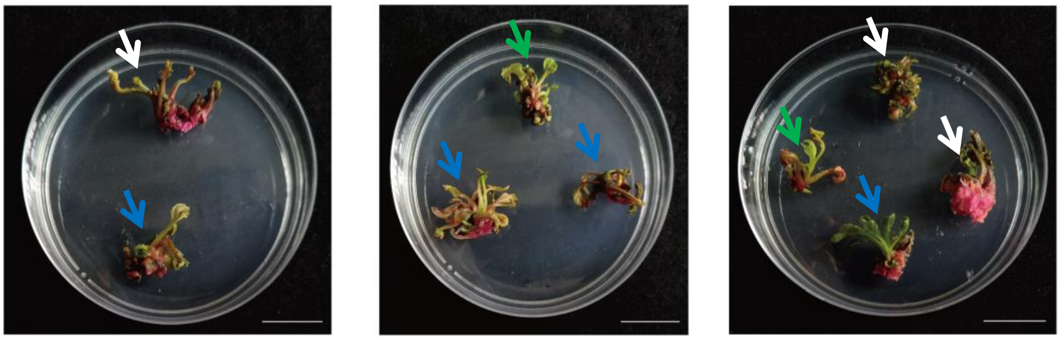

Supplementary Figure S2. The shoots regeneration from red callus in the *A. rhizogenes*-mediated transgenic cv. '49Caixin'. The blue arrows indicate shoots with SAMs; the white arrows indicate abnormal buds; the green arrows indicate green shoots. Scale bar = 20 mm.

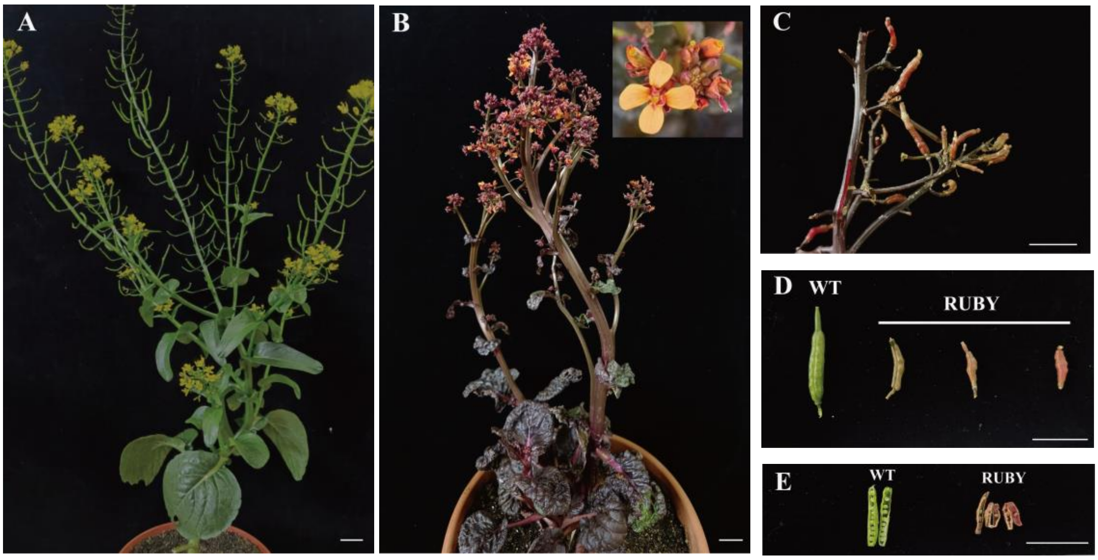

Supplementary Figure S3. The photograph of the representative *A. rhizogenes*-mediated transgenic cv. '49Caixin' overexpressing *RUBY*. WT: cv. '49Caixin' wild type; PAM: protospacer adjacent motif. (A) Mature wild-type cv. '49Caixin' plant. (B) Mature transgenic cv. '49Caixin' plant. (C) Pods produced in the mature transgenic cv. '49Caixin' plant. (D) Comparison of the transgenic cv. '49Caixin' pods with the wild-type pod. (E) Comparison of the transgenic cv. '49Caixin' seeds with the wild-type seeds. Scale bar = 20 mm.

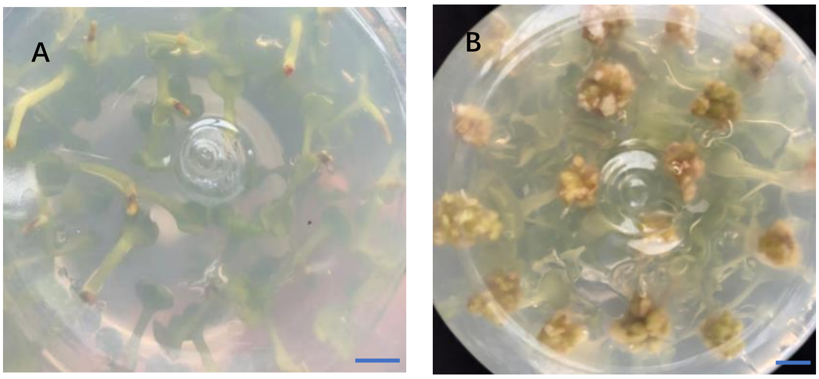

Supplementary Figure S4. The *A. tumefaciens*-mediated transformation of cv. '49Caixin'. (A) The transformed explants cultured on MS medium containing 0.9% agar, 3% sucrose and 200 mg/L carbenicillin. (B) Callus induction on MS medium containing 3% sucrose, 4 mg/L IBA, 3 mg/LNAA, 4 mg/L AgNO3, 0.8% agar and 200 mg/L carbenicillin (pH5.6) showing all the calli were not red and shoots were not regenerated. The images were taken 20 days after transformation. Scale bar = 10 mm.

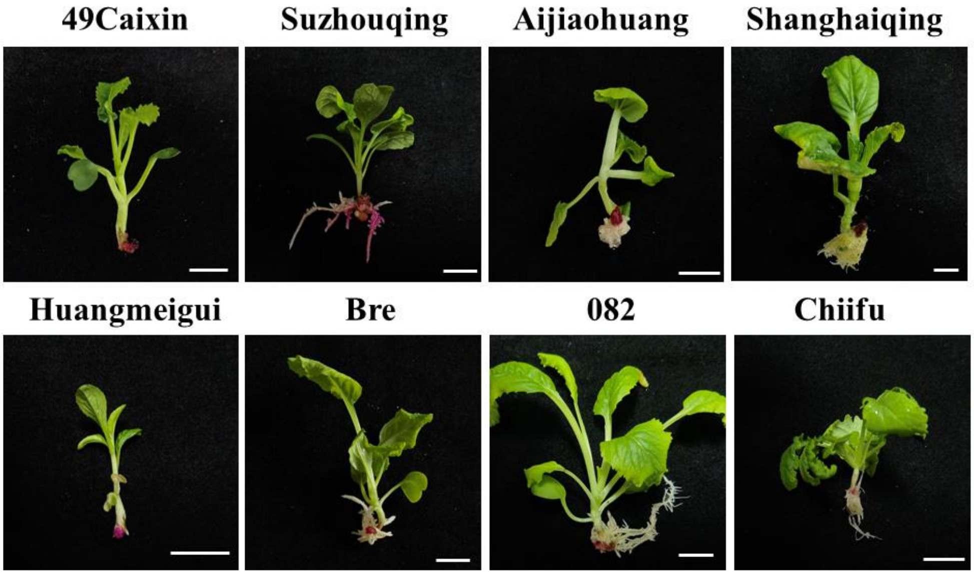

Supplementary Figure S5. Induction of *A. rhizogenes*-mediated transgenic callus in the eight Chinese cabbage cultivars after the hypocotyls were cut off from roots and cultured together with leaves. Scale bar = 20 mm.

>*PDS1* (Bra032770)
5'-
ATGGTTGTGTTTGGGAATGTTTCTGCAGCGAATTTGCCTTATCAAAAT
GGGGTTTTGGAGGCACTTTCATCTGAATTGATGGGACACAGCAGCTTC
AGAGTTCCGATCTCTTCACAAGGGCTTAAGACAAGAACAAGGCGAAG
GACTGCTGGTCCTTTGCAGGTCTCTCTCTGATTCTTGAATTCTTGAAGTTT
TGTATGTGTTTTTTCTTGTGATTAAAGAACACTTTTTTTTGTCCTGGGAGTA
G**GTAGTGTGTGTGGATATACCAAGGCCAGAGCTAGAGAACACTGTCAA**
TTTCTTGGAAGCTGCAAGTTTGTCTGCATCTTTCCGTAGTGCTCCTCG
TCCTGCAAAGCCTTTAAAAGTTGTCATTGCTGGTGCTGGTATGTCATTG
TCCTTTTATTCACTATGCTATCCAATTTGATGTGATGAAAGCTCAAATAAATT
GCAGGATTGGCTGGGCTGTCAACTGCAAAGTACCTGGCCGATGCAGGCCA
CCAACCTCTCTTGCTCAAAGCAAGAGATGTTCTTGGTGGAAAGGTACTTCG
ATTTGTCCATTCACCTAACTCATGAAGTTATTCATTGAGATCATTTACTAACG
AACGAAGATGTGAAATATGTGTGCCTCTAG**ATAGCTGCATGGAAGGATGA**
AGATGGAGATTGGTATGAAACCGGTTTACATATATTTTGTAAGTTCAGAT
TATTAATCTCTCCAATGTGTGTGTTATAAGTTGCTCACATGACGGTTGATATT
TGAAAAATACGGATAGTTTTTACATGTAAAATCTACAAATTTGTGCTTCAGG
CTCTAAGAATCACAGCTAAATAATAATATTTTTTTTAGCAGCATATGTACTTC
TCTTTGCTTAGTGATGATACAGAAAATTCATGATGTCAATGAGAGCATAAGC

ATTTAGAGTTCAATATGATAGTAACCTTTATTATCTTGCACCCCATTTCGAAT
TGTCAGCAAGAAAGATCCCTTTCATAGTTTGAAGGAAGATGTCAATATCATA
CAAAGTTTGTTTTGATTTTCCTAAGTGAAGTTCATAACCTTTTATTTCCTTTT
GACTCGTTTCATATTCTTGCTGGAAG**TCGGTGCTTATCCGAACGTGCAGA**
ACTTATTTGGAGAACTTGGGATTAATGATCGGTTGCAATGGAAGGAAC
ACTCCATGATATTCGCCATGCCAAGTAAACCTGGAGAATTTAGTAGATT
TGATTTCCCAGATGTTCTACCAGCACCCTTAAACGGTATGAGCATAGTCT
TACTCTTATCTGCTAACTAGCTAATATAAATTACTACTTCTAGTTCTTGGAAC
TTTCAAGCATAGAATGTCTGATGAAAACAATGGTTGAATCAAATATGAAAC
ACATGTTCAATAAGATGTATTGAGTAGAGAACTAATCATTTTGCACATTTATA
TGTGTATTGGTCAGTTCACGCCATTGCTATACATCTAATGTAGACTCTGAAA
AGAACATTTTCATTTTTCTTTCCGAGAAAGGAATCTAGTTTTAAGCTTTTAA
TAAACATACGTAAAGATAAGTGTTCACACTATGTAGTTGTAGGCTTGTAGCG
CATAGTGCTGAATTGTCCTCTGTTTGAAATAAATTGTTTTTGTAG**GTATTTG**
GGCAATTTTGAGGAACAACGAGATGCTGACATGGCCAGAGAAAATAAA
GTTTGCTATTGGACTGCTTCCAGCAATGGTCGGAGGTCAGGCTTATGT
TGAGGCCCAAGATGGTTTATCAGTTGAAGAATGGATGAGAAAGCAGG
CAAGTTAGCTTTTTCATTGGTTTGGTTGTGGTTCTTTGTTTCTTCCTTC
TGGTGATATAAATGTTTATTTCCAGGGAGTACCTGATCGCGTGACTGAT
GAAGTGTTTATAGCCATGTCAAAGGCACTTAACTTTATAAACCCGAACG
AACTGTCAATGCAATGCATTTTGATAGCTTTGAACCGGTTTCTTCAGGT
TTGGGCATTCCAATAACTTTTATTCCTCTCTCCATTGACAATATATTGCAATG
CAAATCATAGTTACCACTCAAGCATGCTCACTCCTCATAGAAACTGCTTTGA
CAGGAGAAACATGGTTCAAAGATGGCCTTCTTAGATGGTAATCCTCCGGAG
AGGCTATGCATGCCGATTGTTGAACATATTCGATCGCTAGGTGGCGAAGTAC
GACTCAATTCAAGGATAAGGAAGATTGAACTGGAGGATGATGGTACGGTTA
AGAATTTCTTACTCACTGATGGAACCACTATCCAAGGAGACGCTTATGTGTT
TGCCACTCCAGGTTCTACTTCTTTTCTGAATTTGCATTCCATCTGCTCATTTT
TGTGGCTCCTGTTTACATTTTCTTCTTCTTGTAGTCGATATCCTGAAGCTCCT
TTTGCCGGATTCATGGAAAGAAATACCATACTTCAAGAGACTGGAGAAGTT
AGTTGGTGTTCCAGTCATTAACGTTCATATATGGTTAGTGACAGTCAAGACC
AATACCTGTCATCCTCTTCTGCCATTTTGGTCTCAAGTGCTGTGAATCTATAT
TGCAG**GTTCGATCGGAAACTGAAGAACACATATGATCACTTGCTCTTTA**
GCAGGTGAATAACTTCTCTGAACAATTCAATGGCATTATTCATATTACTTAG
TTTGAACATCCATTAGTAATACTTAAGACGTCTTCATCAAGCAAGAAAATGA
TAAAGATTAGTCTTGAACCACCATAGAAATGAATCAGCAAACAGTGAAGGC
TTGTTCTTGGTTAATTTCTAACAATCTTGTCTTCTTGTTTTGTTTTGCAG**AAG**
TAACCTTCTGAGTGTGTATGCAGACATGTCGTTAACTTGTAAGGTAAA
GAAAGCTAGTCTTGTCGAGTTAATTAGTTGTGTTTCCTTTACTTGATAG
TTTTCTTCTATTTTTCTTAACGCTGTAGGAATATTACGATCCAAACCGAT
CAATGCTGGAGCTAGTATTTGCACCTGCAGAGGAATGGATATCACGGA
CTGACTCTGACATCATCGATGCAACAATGAAAGAGCTAGAGAAAATCT
TCCCTGACGAAATAGCAGCTGACCAAAGCAAAGCTAAAATTCTCAAGT

ACCATGTCGTCAAAACTCCAAGGTTAGACCAAACTCCAAATGTCCCAT
TTAATCTAGTTTCAAATGGATACAATCTCTCTCCTTCTCTCTCTCTATCA
GGTCCGTGTACAAGACCATCCCAGACTGTGAACCATGTCGTCCTCTAC
AGAGATCTCCTATTCAAGGCTTTTACTTAGCTGGAGACTACACTAAACA
GAAGTACTTAGCTTCCATGGAAGGCGCCGTTCTCTCTGGCAAATTCTG
CTCACAGTCTATTGTGCAGGTATATAACACACTAACTTGCCCATTCAAAAG
CATATGTACATCTATTTGTTAACCAGTCACCCTGAATTGTTAAATGATCCGGT
TTTACCAAGTTCGGTCTGTTGATTAGATCTTATGCCACCATTAATCTGTTCCG
GTTACATAGAGATATTGGTCAAGTTTGGGTACACTGATTATACAG**GATTAT**
GAGCTATTGGCTTCCTCTGGACGCCGAAACTTGTCGGAGACAACTGTA
TCAACATGA

>*PDS2* (Bra010751)
5’-
ATGGTTGTGTTTGGGAATGTTTCCGCGGCGAATTTGCCTTATCAAAAT
GGATTTTTGAAGGCAATTTCATCTGGAGGTTGTGATTTAATGGGACAC
CGCAGCTTCAAAATTTCAACTTCTTTTAAGACAAGAACAAGGAGGAGG
AGGAGTGCTGGTCCTTTGCAGGTCTCTGTCTGTTTCATTCAAAAAAAT
CTGATCAAAGTATTATTGGTGATCAAAAGAGCCCAATTTTGAACTGTGT
TGTTAGGTAGTTTGTGTGGATATACCAAGGCCAGAGCTAGAGAACACT
GTCAACTTCTTGGAAGCTGCAAGTTTGTCTGCATCTTTCCGTAGTGCT
CCTCGTCCTGCGAAGCCTTTAAAAGTTGTCATCGCTGGTGCTGGTATG
ATGAATGTGTTTAAACTTATTAGCTTCCTCTTCTTGCTCCCCTTCGGATTCTG
ATGTGGTGAAGCTCTTTTGCCTCTCGAAATAAATTGCAG**GATTGGCTGGAT**
TGTCAACAGCAAAGTACCTGGCTGATGCAGGACATAAACCTCTGTTGC
TTGAAGCGAGAGATGTTCTTGGGGGAAAGGTAAAACTTGTTTACATTCA
TCATTCTTATTAAGACTTGCTAGTCTCCAAAGTCCCTGCACCAATTTGAGTT
TATGAGACTATACTACATGATATATAACTAATGTATAGAGTGTTACAGCTCTT
AGGTACAAAAATTACGTACCTGTATCTGTCTTATTTGTTGTTTTAGTCCTTCA
ATGCCAATTAAAGATCATCTATTAACGATGTGGAAGTATGTGTTCCTCTCCA
G**ATAGCTGCATGGAAGGATGAAGATGGAGATTGGTATGAAACCGGTTT**
ACATATATTTTGTAAGTTCAAAAATTCATACTCTCTCCGTTGCTCCTCCAAG
TATGTATGTATTTTCTCACTTTCATAACTTGGATTTGGAGTTACATGTGATTTA
CCAATATGTGCCCTAGCCTCTTATCTTCTATAGCTGAATTAGATATCTTTTTTA
GTAGCATGCATTTACTGTTAAACGATGTCAGTGAGAGAATAAGCAATTAGA
GTTCAATTTGATAGTCTCAAGGATTCTTGAACCTTTTATAATCTTGCACACCT
TTTCAAATATGCTCATTGGGAAAGATGTAACACAAAGCGTGAGATTAACTA
ATGGTCAACAAAGTTTATTTATCTTTCCTGTTAATTCTATTCCTAATTGAAATT
TAACTCTCTCTTTTTTTCCTTGATTTTTAAAG**TTGGTGCTTATCCGAATGT**
GCAAAACTTATTTGGAGAACTTGGGATCAATGATCGGCTGCAGTGGAA
GGAACACTCCATGATTTTCGCCATGCCAAGTAAACCTGGAGAATTTAG
TAGATTTGATTTCCCAGATGTCCTCCCAGCACCCTTAAACGGTGAGATG
ATAAAACTTATAAGCATAGTTTTTATCTTCAAGTTGTTTGAACCTTTCAAACT

ATATCATATGTTAGGACAACACATGTTGGTTTAAGTTGTTAATGCGGATACTT
TTACCTTTGTAG**GAATATGGGCTATATTGAGGAACAACGAGATGCTGAC**
A**TGGCCAGAGAAAATAAAGTTTGCTATTGGAGTTCTTCCGGCTATGGG**
CGGAGGGCAGGCTTATGTTGAGGCTCGAGATGGTTTATCAGTTGAACA
ATGGATGAGAAAGCAGGCGAGTTCCCTCTGATAGAGTTTCTTTGTTGCTTC
CTTCTCGTGTGCTCAAGTTTTCACTTTGGTTTCTATGTATTCTTTACAG**GGA**
GTACCTGATCGGGTGACAGATGAGGTGTTCATTGCCATGTCAAAGGCG
CTAAACTTTATAAACCCAGACGAACTTTCAATGCAATGCATTTTGATAG
CTTTGAATCGGTTTCTTCAGGTTTTTTTGGCAAACTCTTCCTTTTTCCT
CTCTCTCTCTATTACTCTTCTCTTAATTATAGAAACTGCGATTTTGACAG
GAGAAACATGGATCGAAGATGGCGTTCTTAGATGGTAATCCACCGGAG
AGGCTTTGTATGCCAATAGTGGAACATATTCGATCACTAGGTGGTGAA
GTACGTCTTAACTCAAGGATAAGGAAGATTGAGCTCGAGGATGATGGT
ACTGTTAAGAGTTTCTTACTCACTGATGGAACCACTATCCAAGGAGAC
GCTTATGTCTTTGCCACTCCAGGTTTCATTTCTTGTGCTGTCTTTTTTGGA
TTTACATTCCATTTAAGGGATCCTACTTCATATGCTGTTTTACTCTTGTAG**TG**
GATATCCTGAAGCTTCTTTTGCCGGACTCTTGGAAAGAGATACCATACT
TCAAGAGATTGGAGAAGCTAGTTGGTGTGCCAGTTATTAACGTTCATA
TATGGTTTGATAAGAAACTGAAGAACACATATGATCATTTACTATTTAG
CAGGTGAGTTTCGTGGGAATTTTAAAGGCATCTATGTTCATATTATCCT
CCGTTAATACTTTTGACTTTAAAACTAGTATTCAACAAATCTTGTTGTC
TTTGTGTTGCAGAAGTAACCTTCTGAGTGTGTATGCTGACATGTCGTT
AACGTGTAAGGAATATTACGATCCTAACCGGTCAATGCTGGAGCTAGT
ATTTGCACCTGCGGAGGAATGGATATCAAGGAGCGACTCTGACATCAT
TGATGCAACGATGAAAGAGCTCGAAAGACTCTTCCCTGACGAAATCGC
CGCTGACCAAAGCAAGGCTAAAATTCTCAAGTACCATGTCGTCAAAAC
TCCAAGGTTAGTTAGAAGAAAAGTTATTATACTAAACCCCAAATGTCTCCC
ATTAAAATGATGATGGATACAATATCTATATCTCTATCAG**GTCTGTGTACAA**
GACGATCCCAGACTGTGAACCATGTCGGCCACTACAAAGATCTCCTAT
TAAAGGATTCTACTTAGCTGGGGATTACACTAAACAGAAGTACTTAGCT
TCCATGGAAGGCGCCGTTCTCTCTGGCAAATTCTGCTCTCAGTCTATT
CTACAGGTCAGTCAAAACACATAGTAGATCAGTTTGTTGTTAACCAGTGAC
TCTTTTTCTGGTAAATGATCCGGTTTATTGCTTAGTCCCCCAATTTTTCAAGT
TTGGTTAACTGAACTGATTCTTCAG**GATTACGAGCTATTGGCTGCTTCCT**
CTGGACCGCAAAAGTTGTCGGAGACGACTCTATCAACATAA

Supplementary Figure S6. The genomic DNA sequences of the *PDS1* (*Bra032770*) and *PDS2* (*Bra010751*) genes in the *B. rapa* reference genome (cv. Chiifu V1.5).

Bold, exons; Unbold, introns; Blue, gRNA1. Green, gRNA2. Yellow highlight, PAM

(NGG).

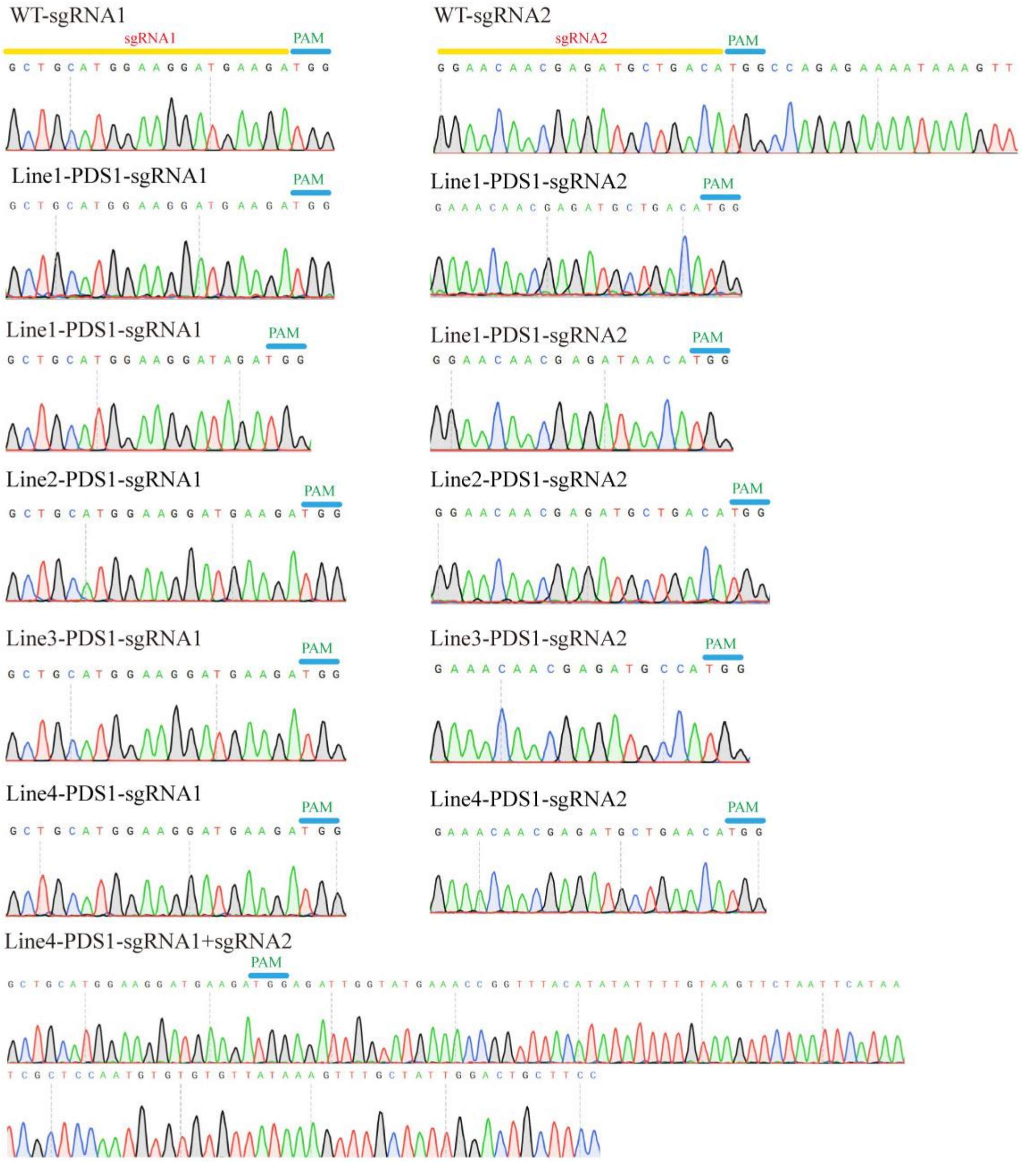

Supplementary Figure S7. Confirming the editing-induced mutations at the two gRNA target sties in the *PDS1* gene in the four albino edited plants (Lines #1 – #4) of cv. ‘49Caixin’. WT, cv. '49Caixin' wild type; PAM, protospacer adjacent motif.

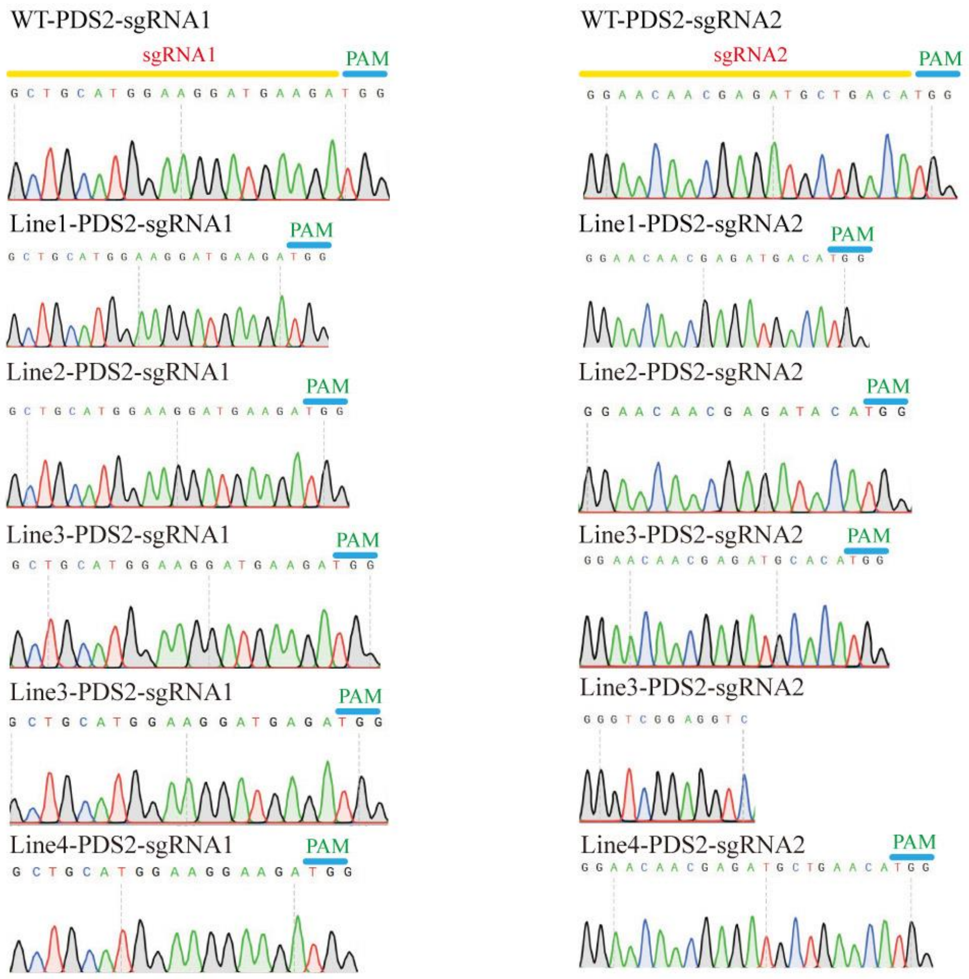

Supplementary Figure S8. Confirming the editing-induced mutations at the two gRNA target sties in the *PDS2* gene in the four albino edited plants (Lines #1 – 4) of cv. '49Caixin'. WT, cv. '49Caixin' wild type; PAM, protospacer adjacent motif.
