# Supplementary material for: Efficient genetic transformation and gene editing of Chinese cabbage using Agrobacterium rhizogenes
Source: Plant Physiol. 2024 Oct 15;197(2):kiae543. doi: 10.1093/plphys/kiae543 (PMC11849774; doi:10.1093/plphys/kiae543)
Supplement: kiae543_Supplementary_Data [file kiae543_supplementary_data.zip › Supplementary Materials and Methods.docx]

**Supplementary Materials and Methods**

**Plant materials and growth conditions**

For the transformation experiments, seeds of 8 Chinese cabbage (*Brassica rapa*) cultivars (‘49Caixin’, ‘Suzhouqing’, ‘Aijiaohuang’, ‘Shanghaiqing’, and ‘Huangmeigui’, ‘Bre’, ‘082’, and ‘Chiifu’) were sterilized using 75% (v/v) alcohol for 3 minutes and 10% (v/v) NaClO for 10 minutes, followed by being washed for 5-6 times using sterile water. The washed seeds were placed on sterilized filter paper and dried. Then, the seeds were placed in 1/2 Murashige and Skoog (1/2 MS) solid medium (pH 5.8) containing 0.9% (w/v) agar and 3% (w/v) sucrose, and cultured under a photoperiod of 16 h light and 8 h dark (Chen et al., 2023). Plants growing in the soil were cultivated in a greenhouse with similar temperature and light conditions.

**Plasmid constructs**

The vector backbone was adopted from the plasmid *35S:RUBY,* which shared by Dr. Yun-De Zhao and Dr. Yu-Bing He. The *35S:ZmWUS2-P2A-IPT-P2A-AtPLT5* fragment containing *ZmWUS2* (GenBank: MP934945.1), *IPT* (GenBank: U83986.1) and *AtPLT5* (AT5G57390) (Wang et al., 2023) was synthesized by Genscript Biotechnology Co., Ltd, and used to replace the *35S:HygR* in *35S:RUBY* with the help of *Xho*I. The resulting plasmid was named WIP-RUBY. The ZmUbi-Cas9-NosT fragment was amplified from pYLCRISPRCas9Pubi-B plasmid (Addgene plasmid #66188) and inserted into the WIP-RUBY plasmid with the help of *Xma*I and *Asc*I. The two gRNAs targeting the coding sequences of *PDS1* (*Bra032770*) and *PDS2* (*Bra010751*) in Chinese cabbage were designed using CRISPR-P 2.0 (<http://crispr.hzau.edu.cn/CRISPR2/>) and cloned into the WIP-RUBY plasmid containing *ZmUbi:Cas9*. The resulting plasmid was name WIP-RUBY-Cas9/gRNA All the sequence information is listed below as **Supplementary sequences**.

***Agrobacterium*-mediated transformation**

The resultant WIP-RUBY-Cas9/gRNA plasmid was confirmed by Sanger sequencing and introduced into *A. tumefaciens* (GV3101) and *A. rhizogenes* (K599). The PCR-validated *Agrobacterium* colonies were added to 200 μl to 30 mL of liquid LB medium containing 50 mg/L kanamycin and 50 mg/L rifampicin or 50 mg/L straptomycin, and incubated in a shaking incubator at 28 °C for 14-16 h. The suspension of *Agrobacterium* was centrifuged at 8000 rpm for 5 min at room temperature to obtain pellet. The pellet was completely resuspended in MS liquid medium and the OD_600_ was adjusted to about 0.8. The hypocotyl explants, which were still attached to cotyledons (Supplementary Figure 1C), were co-cultivated with a suspension of *Agrobacterium* for 10 min. The explants were washed in sterile water and transferred to the MS solid medium (pH 5.8) containing 0.9% (w/v) agar, 3% (w/v) sucrose, and 200 mg/L carbenicillin for *A. rhizogenes* (K599), and MS medium containing 3% (w/v) sucrose, 4 mg/L indole-3-butanoic acid (IBA), 3 mg/L 1-Naphthaleneacetic acid (NAA), 4 mg/L AgNO3, 0.8% (w/v) agar, and 200 mg/L carbenicillin (pH5.6) for *A. tumefaciens* (GV3101) (Lee et al., 2023) and cultured under a photoperiod of 16 h light and 8 h dark. The temperature of the photocell is controlled at 25 °C and the humidity at 50%. After 3 weeks of culture, the number of positive calli tissues of the explants was counted. Then, the calli were cultured to obtain transgenic shoots, which were transferred to the root induction media, i.e., MS media (pH 5.8) containing 0.9% (w/v) agar, 3% (w/v) sucrose, 200 mg/L carbenicillin, and 0.1 mg/L NAA. Transplanted to the soil when regenerated shoots were strong. The regenerated plants were grown in a greenhouse maintained at 24 °C and 16 h light and 8 h dark.

**DNA extraction and PCR identification**

We extracted DNA from the regenereated plants using the Tianamp genomic DNA Kit produced by Tengen. The presence of the *Cas9* transgene was confirmed by PCR using the transgene-specific primers (cas9-F and cas9-R) listed in Supplementary Table S1. The length of the PCR product was 1,716 bp (Supplementary Figure S1).

**Detection of mutation sites**

Primers Bra032770-F, Bra032770-R, Bra010751-F, and Bra010751-R were designed (Supplementary Table S1) to detect mutations in the gene edited lines via the CRISPR website (http://skl.scau.edu.cn/). Firstly, DNA from albino plants was extracted for PCR amplification using primer pairs Bra032770-F / Bra032770-R, and Bra010751-F / Bra010751-R, followed by Sanger sequencing (Lee et al., 2023). The PCR product showing double peaks near the target site were cloned into the pEASY-Blunt cloning vector (TransGen Biotech) for Sanger sequencing (Wang et al., 2023). Finally, multiple sequence comparisons of the wild-type and mutant sequences were performed using DNAMAN (https://www.lynnon.com/) to collate different types of edits.

**References**

**Chen X, Wu Y, Yu Z, Gao Z, Ding Q, Shah SHA, Lin W, Li Y, Hou X.** BcMYB111 responds to BcCBF2 and induces flavonol biosynthesis to enhance tolerance under cold stress in non-heading Chinese cabbage. Int J Mol Sci. 2023:**24**(10): 8670. https://doi.org/10.3390/ijms24108670

**Wang Y, Song S, Hao Y, Chen C, Ou X, He B, Zhang J, Jiang Z, Li C, Zhang S, et al.** Role of BraRGL1 in regulation of *Brassica rapa* bolting and flowering. Hortic Res. 2023:**10**(8):uhad119. https://doi.org/10.1093/hr/uhad119

**Wang D, Zhong Y, Feng B, Qi X, Yan T, Liu J, Guo S, Wang Y, Liu Z, Cheng D, et al.** The RUBY reporter enables efficient haploid identification in maize and tomato. Plant Biotechnol J. 2023:**21**(8):1707-1715.

https://doi.org/10.1111/pbi.14071

**Lee YR, Siddique MI, Kim DS, Lee ES, Han K, Kim SG, Lee HE.** CRISPR/Cas9-mediated gene editing to confer turnip mosaic virus (TuMV) resistance in Chinese cabbage (*Brassica rapa*). Hortic Res. 2023:**10**(6):uhad078. https://doi.org/10.1093/hr/uhad078

**Supplementary sequences**

**The sequences of 35S-ZmWUS2-P2A-IPT-P2A-AtPLT5-NosT**

Red, the *35S* promoter. Orange, *ZmWUS.* Blue, *IPT.* Green, *PLT5*. Purple, P2A. Black, the Nos terminator.

5’-tgagacttttcaacaaagggtaatatcgggaaacctcctcggattccattgcccagctatctgtcacttcatcaaaaggacagtagaaaaggaaggtggcacctacaaatgccatcattgcgataaaggaaaggctatcgttcaagatgcctctgccgacagtggtcccaaagatggacccccacccacgaggagcatcgtggaaaaagaagacgttccaaccacgtcttcaaagcaagtggattgatgtgataacatggtggagcacgacactctcgtctactccaagaatatcaaagatacagtctcagaagaccaaagggctattgagacttttcaacaaagggtaatatcgggaaacctcctcggattccattgcccagctatctgtcacttcatcaaaaggacagtagaaaaggaaggtggcacctacaaatgccatcattgcgataaaggaaaggctatcgttcaagatgcctctgccgacagtggtcccaaagatggacccccacccacgaggagcatcgtggaaaaagaagacgttccaaccacgtcttcaaagcaagtggattgatgtgatatctccactgacgtaagggatgacgcacaatcccactatccttcgcaagaccttcctctatataaggaagttcatttcatttggagaggacacgctgaaatcaccagtctctctctacaaatctatctctctcgagatggcggccaatgcgggcggcggtggagcgggaggaggcagcggcagcggcagcgtggctgcgccggcggtgtgccgccccagcggctcgcggtggacgccgacgccggagcagatcaggatgctgaaggagctctactacggctgcggcatccggtcgcccagctcggagcagatccagcgcatcaccgccatgctgcggcagcacggcaagatcgagggcaagaacgtcttctactggttccagaaccacaaggcccgcgagcgccagaagcgccgcctcaccagcctcgacgtcaacgtgcccgccgccggcgcggccgacgccaccaccagccaactcggcgtcctctcgctgtcgtcgccgccgccttcaggcgcggcgcctccctcgcccaccctcggcttctacgccgccggcaatggcggcggatcggctgtgctgctggacacgagttccgactggggcagcagcggcgctgctatggccaccgagacatgcttcctgcaggactacatgggcgtgacggacacgggcagctcgtcgcagtggccacgcttctcgtcgtcggacacgataatggcggcggccgcggcgcgggcggcgacgacgcgggcgcccgagacgctccctctcttcccgacctgcggcgacgacggcggcagcggtagcagcagctacttgccgttctggggtgccgcgtccacaactgccggcgccacttcttccgttgcgatccaacagcaacaccagctgcaggagcagtacagcttttacagcaacagcaacagcacccagctggccggcaccggcaaccaagacgtatcggcaacagcagcagcagccgccgccctggagctgagcctcagctcatggtgctccccttaccctgctgcagggagtatgggaagcggaGCTACTAACTTCAGCCTGCTGAAGCAGGCTGGAGACGTGGAGGAGAACCCTGGACCTatggatctgcgtctaattttcggtccaacttgcacaggaaagacgtcgaccgcgatacgtcttgcccagcagactggccttccagtcctttcgctcgatcgggtccaatgctgtcctcaactgtcaaccggaagcggacgaccaacagtggaagaactgaaaggaacgacccgtctataccttgaagatcggcctctggtgaagggtatcatcgcagccaagcaagctcacgaaaggctgatcggggaagtgtacaattatgaggcccacggcgggcttattcttgagggaggatctatctcgttgctcaggtgcatggcgcaaagcagttattggagtaccgattttcgttggcatattattcgccacaagttagcagacgaggagacattcatgaacgcggccaaggccagagttaggcagatgttgcgccctgctgtaggcccatctattattcaagagttggttcatctttggaatgagcctcggctgaggcccatactgaaagagatcgacggatatcgatatgccatgttatttgctagccagaaccagatcacacccgatatgctattgcagcttgacccagatatggagggtgagttgattcatggaatcgctcaggagtatctcatccatgcgcgccggcaggagcaggagttccctccagtgagcgtggtcgctttcgaaggattcgaaggtccaccgttcggaatgtgcggaagcggaGCTACTAACTTCAGCCTGCTGAAGCAGGCTGGAGACGTGGAGGAGAACCCTGGACCTATGAAGAACAATAACAACAAATCTTCTTCTTCTTCTAGCTATGATTCTTCTTTGTCTCCTTCTTCTTCATCCTCCTCCCACCAGAACTGGCTCTCTTTCTCTCTCTCCAACAATAACAACAACTTCAATTCTTCCTCAAACCCTAATCTCACTTCCTCCACATCAGATCATCATCATCCTCACCCTTCTCACCTCTCTCTCTTTCAAGCTTTCTCCACTTCTCCAGTCGAACGGCAAGATGGGTCACCGGGAGTTTCACCCAGCGATGCCACGGCGGTTCTTTCCGTATACCCCGGCGGTCCTAAACTTGAGAACTTCCTCGGCGGAGGAGCCTCAACGACGACAACAAGACCAATGCAACAAGTGCAATCTCTTGGCGGCGTTGTCTTCTCTTCCGACCTACAGCCACCGCTTCATCCTCCGTCCGCCGCCGAGATCTACGACTCTGAGCTCAAGTCAATAGCCGCTAGCTTCCTAGGAAACTACTCCGGTGGACACTCGTCGGAGGTCTCTAGCGTACATAAACAACAACCGAATCCTCTAGCTGTCTCAGAGGCTTCGCCTACTCCGAAGAAGAACGTAGAGAGTTTTGGACAACGTACCTCGATTTATAGAGGAGTCACAAGACATAGATGGACTGGAAGATACGAAGCTCATCTATGGGATAATAGTTGCCGAAGAGAAGGCCAAAGCAGAAAAGGAAGACAAGTTTATTTAGGTGGTTATGATAAGGAAGATAAAGCAGCTAGAGCTTACGACCTTGCAGCTCTTAAGTATTGGGGTCCTACAACTACGACTAATTTCCCGATATCAAATTACGAATCTGAACTTGAAGAAATGAAACACATGACTCGACAAGAGTTCGTTGCTTCTTTAAGACGGAAAAGCAGTGGATTCTCTAGGGGTGCCTCCATGTACAGAGGCGTCACTAGACATCATCAGCATGGTCGATGGCAGGCACGAATTGGAAGAGTTGCAGGCAACAAAGACCTTTATCTTGGCACATTTAGCACTCAAGAGGAAGCTGCAGAAGCTTATGATATAGCAGCGATCAAATTCCGCGGTCTAAATGCAGTCACCAATTTCGACATCAGTCGATATGATGTCAAATCAATTGCTAGCTGTAATCTCCCTGTGGGTGGACTAATGCCTAAACCTTCTCCAGCAACCGCAGCGGCTGACAAAACCGTTGATCTTTCTCCATCCGACTCTCCATCTCTAACCACACCGTCCCTCACGTTCAATGTGGCAACACCGGTCAATGACCATGGAGGAACTTTTTACCACACTGGTATACCAATCAAACCAGACCCGGCTGATCATTATTGGTCCAACATCTTTGGATTCCAGGCAAACCCGAAAGCAGAAATGCGACCATTAGCAAACTTTGGGTCGGATCTTCATAACCCTTCTCCTGGTTATGCTATAATGCCGGTAATGCAGGAAGGTGAAAACAACTTTGGTGGTAGTTTTGTTGGGTCTGATGGGTATAACAATCATTCCGCTGCATCGAACCCGGTCTCAGCAATTCCGCTGTCCTCGACAACTACAATGAGTAACGGTAACGAAGGGTATGGTGGAAACATAAACTGGATTAATAACAACATTTCAAGTTCTTACCAAACTGCAAAATCAAATCTCTCTGTTTTGCACACACCGGTTTTTGGGTTGGAATAGctcgagtttctccataataatgtgtgagtagttcccagataagggaattagggttcctatagggtttcgctcatgtgttgagcatataagaaacccttagtatgtatttgtatttgtaaaatacttctatcaataaaatttctaattcctaaaaccaaaatccagtactaaaatccagatc

**The sequence of the ZmUbi-Cas9-NosT**

Red, the *ZmUbi* promoter. Blue, the cas9. Green, the NosT terminator.

5’-GTCGTGCCCCTCTCTAGAGATAATGAGCATTGCATGTCTAAGTTATAAAAAATTACCACATATTTTTTTTGTCACACTTGTTTGAAGTGCAGTTTATCTATCTTTATACATATATTTAAACTTTACTCTACGAATAATATAATCTATAGTACTACAATAATATCAGTGTTTTAGAGAATCATATAAATGAACAGTTAGACATGGTCTAAAGGACAATTGAGTATTTTGACAACAGGACTCTACAGTTTTATCTTTTTAGTGTGCATGTGTTCTCCTTTTTTTTTGCAAATAGCTTCACCTATATAATACTTCATCCATTTTATTAGTACATCCATTTAGGGTTTAGGGTTAATGGTTTTTATAGACTAATTTTTTTAGTACATCTATTTTATTCTATTTTAGCCTCTAAATTAAGAAAACTAAAACTCTATTTTAGTTTTTTTATTTAATAATTTAGATATAAAATAGAATAAAATAAAGTGACTAAAAATTAAACAAATACCCTTTAAGAAATTAAAAAAACTAAGGAAACATTTTTCTTGTTTCGAGTAGATAATGCCAGCCTGTTAAACGCCGTCGACGAGTCTAACGGACACCAACCAGCGAACCAGCAGCGTCGCGTCGGGCCAAGCGAAGCAGACGGCACGGCATCTCTGTCGCTGCCTCTGGACCCCTCTCGAGAGTTCCGCTCCACCGTTGGACTTGCTCCGCTGTCGGCATCCAGAAATTGCGTGGCGGAGCGGCAGACGTGAGCCGGCACGGCAGGCGGCCTCCTCCTCCTCTCACGGCACCGGCAGCTACGGGGGATTCCTTTCCCACCGCTCCTTCGCTTTCCCTTCCTCGCCCGCCGTAATAAATAGACACCCCCTCCACACCCTCTTTCCCCAACCTCGTGTTGTTCGGAGCGCACACACACACAACCAGATCTCCCCCAAATCCACCCGTCGGCACCTCCGCTTCAAGGTACGCCGCTCGTCCTCCCCCCCCCCCCTCTCTACCTTCTCTAGATCGGCGTTCCGGTCCATGGTTAGGGCCCGGTAGTTCTACTTCTGTTCATGTTTGTGTTAGATCCGTGTTTGTGTTAGATCCGTGCTGCTAGCGTTCGTACACGGATGCGACCTGTACGTCAGACACGTTCTGATTGCTAACTTGCCAGTGTTTCTCTTTGGGGAATCCTGGGATGGCTCTAGCCGTTCCGCAGACGGGATCGATTTCATGATTTTTTTTGTTTCGTTGCATAGGGTTTGGTTTGCCCTTTTCCTTTATTTCAATATATGCCGTGCACTTGTTTGTCGGGTCATCTTTTCATGCTTTTTTTTGTCTTGGTTGTGATGATGTGGTCTGGTTGGGCGGTCGTTCTAGATCGGAGTAGAATTCTGTTTCAAACTACCTGGTGGATTTATTAATTTTGGATCTGTATGTGTGTGCCATACATATTCATAGTTACGAATTGAAGATGATGGATGGAAATATCGATCTAGGATAGGTATACATGTTGATGCGGGTTTTACTGATGCATATACAGAGATGCTTTTTGTTCGCTTGGTTGTGATGATGTGGTGTGGTTGGGCGGTCGTTCATTCGTTCTAGATCGGAGTAGAATACTGTTTCAAACTACCTGGTGTATTTATTAATTTTGGAACTGTATGTGTGTGTCATACATCTTCATAGTTACGAGTTTAAGATGGATGGAAATATCGATCTAGGATAGGTATACATGTTGATGTGGGTTTTACTGATGCATATACATGATGGCATATGCAGCATCTATTCATATGCTCTAACCTTGAGTACCTATCTATTATAATAAACAAGTATGTTTTATAATTATTTTGATCTTGATATACTTGGATGATGGCATATGCAGCAGCTATATGTGGATTTTTTTAGCCCTGCCTTCATACGCTATTTATTTGCTTGGTACTGTTTCTTTTGTCGATGCTCACCCTGTTGTTTGGTGTTACTTCTGCAG

ATGGCTCCTAAGAAGAAGCGGAAGGTTGGTATTCACGGGGTGCCTGCGGCTGACAAGAAGTACTCCATCGGCCTCGACATCGGCACCAACAGCGTCGGCTGGGCGGTGATCACCGACGAGTACAAGGTCCCGTCCAAGAAGTTCAAGGTCCTGGGCAACACCGACCGCCACTCCATCAAGAAGAACCTCATCGGCGCCCTCCTCTTCGACTCCGGCGAGACGGCGGAGGCGACCCGCCTCAAGCGCACCGCCCGCCGCCGCTACACCCGCCGCAAGAACCGCATCTGCTACCTCCAGGAGATCTTCTCCAACGAGATGGCGAAGGTCGACGACTCCTTCTTCCACCGCCTCGAGGAGTCCTTCCTCGTGGAGGAGGACAAGAAGCACGAGCGCCACCCCATCTTCGGCAACATCGTCGACGAGGTCGCCTACCACGAGAAGTACCCCACTATCTACCACCTTCGTAAGAAGCTTGTTGACTCTACTGATAAGGCTGATCTTCGTCTCATCTACCTTGCTCTCGCTCACATGATCAAGTTCCGTGGTCACTTCCTTATCGAGGGTGACCTTAACCCTGATAACTCCGACGTGGACAAGCTCTTCATCCAGCTCGTCCAGACCTACAACCAGCTCTTCGAGGAGAACCCTATCAACGCTTCCGGTGTCGACGCTAAGGCGATCCTTTCCGCTAGGCTCTCCAAGTCCAGGCGTCTCGAGAACCTCATCGCCCAGCTCCCTGGTGAGAAGAAGAACGGTCTTTTCGGTAACCTCATCGCTCTCTCCCTCGGTCTGACCCCTAACTTCAAGTCCAACTTCGACCTCGCTGAGGACGCTAAGCTTCAGCTCTCCAAGGATACCTACGACGATGATCTCGACAACCTCCTCGCTCAGATTGGAGATCAGTACGCTGATCTCTTCCTTGCTGCTAAGAACCTCTCCGATGCTATCCTCCTTTCGGATATCCTTAGGGTTAACACTGAGATCACTAAGGCTCCTCTTTCTGCTTCCATGATCAAGCGCTACGACGAGCACCACCAGGACCTCACCCTCCTCAAGGCTCTTGTTCGTCAGCAGCTCCCCGAGAAGTACAAGGAGATCTTCTTCGACCAGTCCAAGAACGGCTACGCCGGTTACATTGACGGTGGAGCTAGCCAGGAGGAGTTCTACAAGTTCATCAAGCCAATCCTTGAGAAGATGGATGGTACTGAGGAGCTTCTCGTTAAGCTTAACCGTGAGGACCTCCTTAGGAAGCAGAGGACTTTCGATAACGGCTCTATCCCTCACCAGATCCACCTTGGTGAGCTTCACGCCATCCTTCGTAGGCAGGAGGACTTCTACCCTTTCCTCAAGGACAACCGTGAGAAGATCGAGAAGATCCTTACTTTCCGTATTCCTTACTACGTTGGTCCTCTTGCTCGTGGTAACTCCCGTTTCGCTTGGATGACTAGGAAGTCCGAGGAGACTATCACCCCTTGGAACTTCGAGGAGGTTGTTGACAAGGGTGCTTCCGCCCAGTCCTTCATCGAGCGCATGACCAACTTCGACAAGAACCTCCCCAACGAGAAGGTCCTCCCCAAGCACTCCCTCCTCTACGAGTACTTCACGGTCTACAACGAGCTCACCAAGGTCAAGTACGTCACCGAGGGTATGCGCAAGCCTGCCTTCCTCTCCGGCGAGCAGAAGAAGGCTATCGTTGACCTCCTCTTCAAGACCAACCGCAAGGTCACCGTCAAGCAGCTCAAGGAGGACTACTTCAAGAAGATCGAGTGCTTCGACTCCGTCGAGATCAGCGGCGTTGAGGACCGTTTCAACGCTTCTCTCGGTACCTACCACGATCTCCTCAAGATCATCAAGGACAAGGACTTCCTCGACAACGAGGAGAACGAGGACATCCTCGAGGACATCGTCCTCACTCTTACTCTCTTCGAGGATAGGGAGATGATCGAGGAGAGGCTCAAGACTTACGCTCATCTCTTCGATGACAAGGTTATGAAGCAGCTCAAGCGTCGCCGTTACACCGGTTGGGGTAGGCTCTCCCGCAAGCTCATCAACGGTATCAGGGATAAGCAGAGCGGCAAGACTATCCTCGACTTCCTCAAGTCTGATGGTTTCGCTAACAGGAACTTCATGCAGCTCATCCACGATGACTCTCTTACCTTCAAGGAGGATATTCAGAAGGCTCAGGTGTCCGGTCAGGGCGACTCTCTCCACGAGCACATTGCTAACCTTGCTGGTTCCCCTGCTATCAAGAAGGGCATCCTTCAGACTGTTAAGGTTGTCGATGAGCTTGTCAAGGTTATGGGTCGTCACAAGCCTGAGAACATCGTCATCGAGATGGCTCGTGAGAACCAGACTACCCAGAAGGGTCAGAAGAACTCGAGGGAGCGCATGAAGAGGATTGAGGAGGGTATCAAGGAGCTTGGTTCTCAGATCCTTAAGGAGCACCCTGTCGAGAACACCCAGCTCCAGAACGAGAAGCTCTACCTCTACTACCTCCAGAACGGTAGGGATATGTACGTTGACCAGGAGCTCGACATCAACAGGCTTTCTGACTACGACGTCGACCACATTGTTCCTCAGTCTTTCCTTAAGGATGACTCCATCGACAACAAGGTCCTCACGAGGTCCGACAAGAACAGGGGTAAGTCGGACAACGTCCCTTCCGAGGAGGTTGTCAAGAAGATGAAGAACTACTGGAGGCAGCTTCTCAACGCTAAGCTCATTACCCAGAGGAAGTTCGACAACCTCACGAAGGCTGAGAGGGGTGGCCTTTCCGAGCTTGACAAGGCTGGTTTCATCAAGAGGCAGCTTGTTGAGACGAGGCAGATTACCAAGCACGTTGCTCAGATCCTCGATTCTAGGATGAACACCAAGTACGACGAGAACGACAAGCTCATCCGCGAGGTCAAGGTGATCACCCTCAAGTCCAAGCTCGTCTCCGACTTCCGCAAGGACTTCCAGTTCTACAAGGTCCGCGAGATCAACAACTACCACCACGCTCACGATGCTTACCTTAACGCTGTCGTTGGTACCGCTCTTATCAAGAAGTACCCTAAGCTTGAGTCCGAGTTCGTCTACGGTGACTACAAGGTCTACGACGTTCGTAAGATGATCGCCAAGTCCGAGCAGGAGATCGGCAAGGCCACCGCCAAGTACTTCTTCTACTCCAACATCATGAACTTCTTCAAGACCGAGATCACCCTCGCCAACGGCGAGATCCGCAAGCGCCCTCTTATCGAGACGAACGGTGAGACTGGTGAGATCGTTTGGGACAAGGGTCGCGACTTCGCTACTGTTCGCAAGGTCCTTTCTATGCCTCAGGTTAACATCGTCAAGAAGACCGAGGTCCAGACCGGTGGCTTCTCCAAGGAGTCTATCCTTCCAAAGAGAAACTCGGACAAGCTCATCGCTAGGAAGAAGGATTGGGACCCTAAGAAGTACGGTGGTTTCGACTCCCCTACTGTCGCCTACTCCGTCCTCGTGGTCGCCAAGGTGGAGAAGGGTAAGTCGAAGAAGCTCAAGTCCGTCAAGGAGCTCCTCGGCATCACCATCATGGAGCGCTCCTCCTTCGAGAAGAACCCGATCGACTTCCTCGAGGCCAAGGGCTACAAGGAGGTCAAGAAGGACCTCATCATCAAGCTCCCCAAGTACTCTCTTTTCGAGCTCGAGAACGGTCGTAAGAGGATGCTGGCTTCCGCTGGTGAGCTCCAGAAGGGTAACGAGCTTGCTCTTCCTTCCAAGTACGTGAACTTCCTCTACCTCGCCTCCCACTACGAGAAGCTCAAGGGTTCCCCTGAGGATAACGAGCAGAAGCAGCTCTTCGTGGAGCAGCACAAGCACTACCTCGACGAGATCATCGAGCAGATCTCCGAGTTCTCCAAGCGCGTCATCCTCGCTGACGCTAACCTCGACAAGGTCCTCTCCGCCTACAACAAGCACCGCGACAAGCCCATCCGCGAGCAGGCCGAGAACATCATCCACCTCTTCACGCTCACGAACCTCGGCGCCCCTGCTGCTTTCAAGTACTTCGACACCACCATCGACAGGAAGCGTTACACGTCCACCAAGGAGGTTCTCGACGCTACTCTCATCCACCAGTCCATCACCGGTCTTTACGAGACTCGTATCGACCTTTCCCAGCTTGGTGGTGATAAGCGTCCTGCTGCCACCAAAAAGGCCGGACAGGCTAAGAAAAAGAAGTAGTCCCGATCGTTCAAACATTTGGCAATAAAGTTTCTTAAGATTGAATCCTGTTGCCGGTCTTGCGATGATTATCATATAATTTCTGTTGAATTACGTTAAGCATGTAATAATTAACATGTAATGCATGACGTTATTTATGAGGTGGGTTTTTATGATTAGAGTCCCGCAATTATACATTTAATACGCGATAGAAAACAAAATATAGCGCGCAAACTAGGATAAATTATCGCGCGCGGTGTCATCTATGTTACTAGA

**The sequences of the gRNA-expressing cassettes**

Red, the *AtU6-26* and *AtU6-29* promoters that drive the gRNAs. Blue, the gRNA. Gold, the gRNA scaffold. Green, the terminator.

>Atu6-26: gRNA1

5’-CGTTGAACAACGGAAACTCGACTTGCCTTCCGCACAATACATCATTTCTTCTTAGCTTTTTTTCTTCTTCTTCGTTCATACAGTTTTTTTTTGTTTATCAGCTTACATTTTCTTGAACCGTAGCTTTCGTTTTCTTCTTTTTAACTTTCCATTCGGAGTTTTTGTATCTTGTTTCATAGTTTGTCCCAGGATTAGAATGATTAGGCATCGAACCTTCAAGAATTTGATTGAATAAAACATCTTCATTCTTAAGATATGAAGATAATCTTCAAAAGGCCCCTGGGAATCTGAAAGAAGAGAAGCAGGCCCATTTATATGGGAAAGAACAATAGTATTTCTTATATAGGCCCATTTAAGTTGAAAACAATCTTCAAAAGTCCCACATCGCTTAGATAAGAAAACGAAGCTGAGTTTATATACAGCTAGAGTCGAAGTAGTGATTGGGCTGCATGGAAGGATGAAGAGTTTCAGAGCTATGCTGGAAACAGCATAGCAAGTTGAAATAAGGCTAGTCCGTTATCAACTTGAAAAAGTGGCACCGAGTCGGTGCTTTTTTTGTCCCTTCGAAGGGCCTTTCTCACATCAC

>AtU6-29: gRNA2

5’-ctgatagattgtatatccatgtttagttggattaaatatcaaagatctcttacagttagtttcgttcttaatccaaactactgcagcctgacagacaaatgaggatgcaaacaattttaaagtttatctaacgctagctgttttgtttcttctctctggtgcaccaacgacggcgttttctcaatcataaagaggcttgttttacttaaggccaataatgttgatggatcgaaagaagagggcttttaataaacgagcccgtttaagctgtaaacgatgtcaaaaacatcccacatcgttcagttgaaaatagaagctctgtttatatattggtagagtcgactaagagattGGGAAACAACGAGATGCTGACAGTTTCAGAGCTATGCTGGAAACAGCATAGCAAGTTGAAATAAGGCTAGTCCGTTATCAACTTGAAAAAGTGGCACCGAGTCGGTGCTTTTTTTGTCCCTTCGAAGGGCCTTTCTCACATCAC
